# Rehabilitation service development for Sub-Acute and Non-Acute Patient (SNAP) under universal coverage scheme in Thailand

**DOI:** 10.1186/1472-6963-12-S1-O6

**Published:** 2012-11-21

**Authors:** Orathai Khiaocharoen, Supasit Pannarunothai, Preeda Taearak, Wachara Riewpaiboon, Chairoj Zungsontiporn

**Affiliations:** 1 Phitsanulok Provincial Health Office, Thailand; 2 Centre for Health Equity Monitoring (CHEM) Faculty of Medicine, Naresuan University, Thailand; 3 National Health Security Office Region 8 Udon Thani, Thailand; 4 Health Systems Research Institute (HSRI), Thailand; 5 Central Office for Healthcare Information, Thailand

## 

The need of rehabilitation in sub-acute and non-acute patients has been continuously increased. Rehabilitation services in Thailand are considered only as part of acute care so that the providers are not motivated to provide intensive services to patients because the payment based on diagnosis related group focuses on acute phase. This study aimed to develop an appropriate model for SNAP Casemix including in-patient, out-patient, and home based care in 4 groups of patients: stroke, brain dysfunction (traumatic and non-traumatic), spinal cord dysfunction (traumatic and non-traumatic) and major multiple trauma. The expected results are improvement of accessibility, continuity, quality of care as well as the proper payment and information of activities, unit costs. Fifty five hospitals in five provinces were recruited voluntarily to develop rehabilitation services both facility-based and home-based care, referral system, structure, payment, information system etc. Three development steps were set up as follows: 1) setting the new desirable system, 2) implementation of the new system (according to context of each province) and 3) evaluation. The effectiveness will be assessed through functional status and quality of life gained compared to set target. The efficiency studies consist of cost per patient, cost per DRG and cost recovery. Barthel Index (assessment of 10 functions: feeding, transfer, grooming, toilet use, bathing, mobility, stairs climbing, dressing, bowels, and bladder) and EQ5D (Assessment of quality of life in 5 dimensions including: mobility, self-care, usual activities, pain/discomfort and anxiety/depression) will be used for functional assessment. The study will go on for 18 months.

